# The role of health-based food choice motives in explaining the relationship between lower socioeconomic position and higher BMI in UK and US adults

**DOI:** 10.1038/s41366-022-01190-4

**Published:** 2022-07-21

**Authors:** Eric Robinson, Andrew Jones, Lucile Marty

**Affiliations:** 1 Department of Psychological Sciences, University of Liverpool, Liverpool, UK; 2 Centre des Sciences Du Goût et de l’Alimentation, Agrosup Dijon, CNRS, INRAE, Université Bourgogne Franche-Comté, F-21000 Dijon, France

**Keywords:** Nutrition, Public health

## Abstract

**Background/Objectives:**

Lower socioeconomic position (SEP) is associated with increased risk of higher BMI and developing obesity. No research to date has directly examined whether SEP differences in health-based food choice motives or executive function explain why lower SEP is associated with higher BMI.

**Subjects/Methods:**

We analysed observational data from large samples of UK (*N* = 4130) and US (*N* = 1898) adults which included measures of SEP (education level, household income and subjective social status) and self-reported BMI. Participants also completed validated self-report measures on the extent to which their day-to-day food choices were motivated by health and weight control, as well as completing computerized tasks measuring inhibitory control (Stroop task) and working memory (Digit span task).

**Results:**

Across both UK and US adults, the relationship between indicators of lower SEP and higher BMI were consistently explained by participants from lower SEP backgrounds reporting being less motivated by health when making food choices, which accounted for 18–28% of the association between lower SEP and higher BMI. There was no evidence that measures of executive function explained associations between SEP and BMI or moderated relations between food choice motives and higher BMI.

**Conclusions:**

SEP differences in health-based food choice motives may play an important role in explaining why lower SEP is associated with an increased risk of higher BMI.

## Background

Socioeconomic position (SEP) refers to the social (e.g. level of education) and financial (e.g. household income) factors that determine a person’s position or perceived position in society [1]. Lower SEP tends to be associated with increased risk of higher BMI in developed countries [2, 3]. A range of factors, including SEP differences relating to exposure to unhealthy food outlets and household income likely contribute to this association [4, 5], but psychological factors may also play an important role. Across studies of European adults, there is consistent evidence that lower SEP (i.e. education level) is associated with being less motivated by health when making food choices [6–8]. Other indicators of lower SEP (e.g. household income, occupation type) are associated with food choice motives being less influenced by health [9, 10], but not in all studies [11]. A related but distinct food choice motive is weight control motivation, although there is less research examining the links between measures of SEP, weight control motives and dietary patterns [11]. The extent to which individuals are motivated by health when making dietary decisions is predictive of healthier diet [10, 12] and reduced likelihood of overweight [13]. However, no research has directly examined whether the relationship between lower SEP and higher BMI is explained by SEP differences in food choice motives.

A further psychological factor that may explain SEP differences in BMI is executive function. Executive function is a set of mental processes that allow people to attend to information, plan and monitor behaviour [14]. In the context of obesity, both inhibitory control (e.g. the ability to control impulsive responses, such as desires for unhealthy food) and working memory (e.g. the ability to hold competing information in mind, such as relative healthiness of food vs. sensory appeal) may be important. Lower SEP is associated with reduced executive function [15, 16], while reduced executive function is associated with less healthy diet and higher BMI [15, 17–20], which could in part explain why lower SEP is predictive of higher BMI [21]. A further consideration is that executive function may interact with food choice motives to determine likelihood of maintaining a healthy body weight, as ability to translate motives into long-term behaviour may be dictated by individual differences in executive function [17, 22], but these hypotheses are yet to be tested.

Our primary aim was to directly examine for the first time whether the relationship between lower SEP and higher BMI is in part explained by SEP differences in food choice motives relating to health and weight control in a large sample of UK adults. Consistent with previous research we examined education and income as measures of SEP [6, 8, 11, 23], as well as subjective social status because this may be an additional independent SEP predictor of higher BMI [24]. Measures of executive function in a sub-sample of participants allowed us to explore whether individual differences in executive function explain (i) the link between SEP and higher BMI or (ii) moderate relationships between food choice motives and BMI. Finally, moving beyond existing work in European samples [6–9, 11], we examined cultural generalisability of findings in a sub-sample of US adults.

## Methods

### Participants

We made use of data collected from UK and US adults participating in six online studies that used similar methodology to examine the effect of structural and information-based interventions on simulated dietary choice. In all studies, participants reported on health and weight control food choice motives, SEP indices (education, income, subjective socioeconomic status) and BMI (calculated from self-reported weight and height). Studies received ethical approval from the University of Liverpool Health and Life Sciences Research Ethics Committee and informed consent was obtained from all participants. Participants were recruited online from Prolific Academic (UK participants) or Amazon Mechanical Turk (US participants). Both Prolific Academic and Amazon Mechanical Turk are widely used participant recruitment sites for online survey based research in which members of the general public register and complete online research studies in return for financial compensation. Participants were eligible to participate if they were UK/US residents, aged 18 or above, fluent in English, had access to a computer with an internet connection, and had no dietary restrictions. All studies aimed to recruit a sample stratified by gender and by highest educational qualification to be broadly representative of the UK/US adult population and contain similar numbers of “higher” and “lower” educated adults. Studies 1 and 2 [25] examined dietary choice in a virtual fast food restaurant. UK participants (*n* = 1743 in original studies) selected a meal after being randomized to one of four conditions in a 2 × 2 between-subjects design: menu energy labelling present vs. absent and increased vs. normal availability of lower energy options. Study 3 [26] examined simulated supermarket purchasing. UK participants (*n* = 899) were randomized in a 2 × 2 between-subjects design to: labelling of lower energy density products (vs. absence) and increased (vs. normal) availability of lower energy density products. Study 4 [27] examined hypothetical portion size selection. UK participants (*n* = 1667) selected their desired portion size for main meals in the absence or presence of different types of energy labelling. In studies 5 and 6 [28], US participants (*n* = 2091) made simulated dietary choices from six sit-down restaurant menus after being randomized to: the absence vs. presence of menu energy labelling and from menus with normal vs. increased availability of lower energy main dishes. In all studies, demographic data were collected at the beginning of the study. Food choice motives and executive function measures were collected at the end of the study.

### SEP measures

#### Education level

Participants reported on their highest education level. UK participants completed the following items: “What is your highest educational qualification? If you are a student please select the diploma being studied for.” *No formal qualifications, 1-3 GCSEs or equivalent, 4* + *GCSEs or equivalent, A level or equivalent, Certificate of higher education (CertHE) or equivalent, Diploma of higher education (DipHE) or equivalent, Bachelor’s degree or equivalent, Master’s degree or equivalent, Doctoral degree or equivalent*. Participants also reported on years in higher education using a free-text response format: “After leaving school (i.e. at 16 years old), how many further years of higher education (i.e. a formal course) did you study for?”. US participants completed the following items: “What is your highest educational qualification? If you are a student please select the diploma being studied for.” *Less than high-school, High-school completion, Some college or associate degree, Bachelor’s degree, Master’s degree, Doctoral or professional degree*. “After leaving middle school (i.e. after 8th grade), how many further years of higher education did you study for?” (free-text).

#### Household income

UK participants were asked to report the annual after-tax income of their household including all earners to the nearest £1000. Participants also reported on the number of adults and children (<14 y) living in their household. Equivalised household income was calculated by dividing the after-tax household income by the sum of the equivalence value of all the household members (first adult = 1, additional adult or child aged 14 and over = 0.5, child aged 0–13 = 0.3). US participants reported their annual household income (before tax) to the nearest $1000.

#### Subjective social status

Both UK and US participants rated where they believed they are in society from 1 (people who have the least money, least education and the worst jobs or no job) to 10 (people who have the most money, most education and the best jobs) using the MacArthur scale of subjective social status [29].

#### Food choice motives measures

In studies 1, 2 and 5 participants completed a food choice questionnaire [30] in which the following two statements “It is important to me that the food I eat on a typical day: is healthy” (health motivation) and “It is important to me that the food I eat on a typical day: helps me control my weight” (weight control motivation) were both rated on scales from 1 (Not at all important) to 7 (Very important). The health and weight control motivation items were answered alongside other 1-item dimensions [30]. In studies 3, 4 and 6, participants completed the health and weight control subscales of the Food Choice Questionnaire [31]. The health subscale has 6 items (e.g. “It is important to me that the food I eat on a typical day keeps me healthy”) and weight control subscale has 3 items (e.g. “It is important to me that the food I eat on a typical day helps me control my weight”), with response options for each question ranging from 1 (Not at all important) to 4 (Very important): resulting in mean scale scores ranging from 1 to 4 for the health and weight control motives scales.

#### Executive function measures

In studies 1, 2 and 4, the UK participants completed two measures of executive function. A Stroop task was used to measure inhibitory control. See online supplementary materials for full task information. The Stroop interference effect was calculated as the difference between the median reaction times (RTs) of the incongruent trials and the congruent trials [incongruent RT—congruent RT] for correct trials only. A larger interference score is indicative of poorer inhibition. We also calculated the proportion of correct responses in incongruent trials, as a secondary outcome because there is some evidence of an association with poorer diet [32]. We used a backwards digit-span task to measure working memory. See online supplementary materials for full task information. The primary outcome was the two-error maximum length as the last digit-span a participant got correct before making two consecutive errors and as a secondary outcome we included maximum length i.e., the maximal backward digit span that a participant recalled correctly during all 14 trials.

#### Standardising of variables

To ensure comparability across UK and US studies, we dichotomised highest education level into “lower” (anything below UK degree/US college level) and “higher” (degree/college level and above). To account for both the level of qualification achieved and time spent in education, we calculated a secondary continuous composite measure of amount of education, as the mean of the z-scores for highest educational level and years in higher education for each study. To account for the non-linear distribution of income participants were recoded into quintiles (quintiles calculated for UK and US data separately). To account for the difference in the number of items included in the two scales used to measure food choice motives (i.e. single item measures vs. multi-item measures), in primary analyses we treated health and weight control motives as single item measures (i.e. we used the 1 question from the multi-item scale that was directly comparable to the question from the single item) with data z-scored within studies to account for differences in response scales. To gauge whether results were consistent when multi-item scale scores were available, in sensitivity analyses we z-scored mean scale scores in each study (e.g. to account for different scale scoring for single item vs. multi-item scales). In a further unplanned sensitivity analyses we then repeated the primary analysis on mean scale scores limited to studies in which only the multi-item health and weight control motives scales were collected.

#### Data exclusions

As in the original studies, any participants that failed one or more attention checks (questions included in the studies to detect careless responding [32]) or did not complete the study in full were not included. Because our main interest was in the relationship between food choice motives, SEP and higher BMI, we excluded participants with a BMI < 18.5. In line with [33, 34], we excluded participants with implausible weight (<30 kg or >250 kg) and height (<145 cm or >3 m) values or likely implausible BMI (>70) values. For income data, if a participant reported a household income that was extreme i.e. approximately >10 times the UK median equivalised income [>£300,000] or US median [>$650,000] their data was treated as missing. See online supplementary materials for individual study sample sizes and data exclusions.

#### Analyses

The analysis protocol was pre-registered is available with the study data at https://osf.io/tjgcy/.

#### Primary analyses for SEP, food choice motives and BMI (UK sample)

To examine whether measures of SEP were associated with food choice motives we conducted two linear regression models (z-scored single item health motives and weight control motives as dependent variables), with age, gender, ethnicity (white vs, not), BMI (continuous) household income, highest education level (lower vs. higher) and subjective social status as predictor variables. To test whether food choices motives independently predict BMI we planned a further regression (BMI dependent variable) controlling for the same demographic and each SEP measure. Next, we planned to identify any measures of SEP that were associated with BMI (in unadjusted raw associations). If we found evidence that a measure(s) of SEP was associated with BMI, and that the same measure(s) of SEP was associated with a food choice motivation measure (health and/or weight control motives) that was in turn associated with BMI in regression analyses, we planned to conduct a formal indirect effects analysis to test whether food choices motives mediated SEP-BMI associations. If more than one SEP measure was identified for indirect effects analyses we planned to conduct indirect effect analysis for each and if both health and weight control motives were associated with the same measure of SEP and BMI, we conducted parallel indirect effects analyses to examine their independent indirect effects. In primary analyses alpha was set at 0.05.

#### Secondary analyses for SEP, food choice motives and BMI (US sample)

We replicated the above primary analyses in the US sample.

#### Secondary analyses examining executive function (UK sample)

To explore whether measures of executive function explained associations between measures of SEP and BMI we repeated the above primary analysis strategy, but replaced food choice motive measured with the measures of executive function when predicting BMI. To examine whether the relationship between food choice motives and BMI was moderated by measures of executive function, we conducted linear regression in which we included measures of executive function, food choice motives and mean centred interaction terms between each measure of executive function and food choice motives in a second step of the model. To account for multiple comparisons, for all secondary analyses alpha was set a 0.01. 99% confidence intervals are reported.

#### Sensitivity analyses

We repeated primary and secondary analyses using the secondary composite (continuous) measure of education level, as well as replacing the z-scored single item food motive measures with the z-scored multi-item measures, where available. We also examined if results were consistent when the alternate measures of inhibitory control (Stroop proportion correct as opposed to interference) and working memory (maximum total as opposed to two error total) were used.

#### Sample size

To be powered to detect statistically small unadjusted associations (*r* = 0.10) between variables of interest (GPOWER 3.1.3, 90% power, *p* < 0.01) and statistically small effects in the regression and indirect effects analysis models described above [35], we estimated a minimum sample size of N~1500. Available data for both UK and US participants exceeded this. Analyses were conducted in SPSS25 with the exception of indirect effect analyses that we conducted in SAS using the PROCESS MACRO (MODEL 4).

## Results

### UK sample characteristics

Complete data were available for *N* = 4130 UK (2092/51% female) participants. Of the sample, 47% had an education level that was university degree or higher. The sample’s mean BMI = 27.1 (SD = 5.9) and 57% were classed as having a BMI in the overweight or obesity range. See Table 1 for sample characteristics. Lower household income (*r* = −0.06), lower subjective social status (*r* = −0.14) and lower education level (*r* = −0.11) were significantly associated with higher BMI. Higher BMI was significantly associated with being less motivated by health when making food choices (*r* = −0.12) and more motivated by weight control (*r* = 0.08). See online supplementary material for unadjusted associations between BMI, food choice motives and measures of SEP. Lower household income, lower subjective status and lower education level were all significantly associated with being less motivated by health (*r* = 0.12, *r* = 0.21, *r* = 0.18 respectively) and weight control (*r* = 0.07, *r* = 0.12, *r* = 0.04). For proportions of participants endorsing health and weight control as important food choice motives (vs. not) split by SEP, BMI and demographic categories, see online supplementary material.

**Table 1:** UK and US sample characteristics.

	UK (*N* = 4130)	US (*N* = 1898)
Gender (Female)	2092 (51%)	1041 (55%)
Ethnicity (White)	3785 (92%)	1546 (82%)
Age (M years, SD)	37 (13)	41 (17)
BMI (M, SD)	27.1 (5.9)	28.5 (7.4)
Normal weight BMI	1769 (43%)	729 (38%)
Overweight BMI	1367 (33%)	575 (30%)
Obesity BMI	994 (24%)	594 (31%)
Education level (Higher)	1924 (47%)	1238 (65%)
Household income (M, SD)	£21,163 (£15, 169)	$54, 912 ($45,874)
Subjective social status (9 M, SD)	5.1 (1.6)	4.9 (1.8)
	UK (*N* = 3256)	
Inhibitory control: Stroop interference, (M, SD)	237.5 (238.5)	–
Inhibitory control: Stroop proportion correct (M, SD)	0.90 (0.12)	–
Working memory: Two error maximum length (M, SD)	5.9 (1.8)	–
Working memory: Maximum length (M, SD)	6.7 (1.7)	–

Education level (Higher)denotes degree/college level and above. Household income is equivalised for UK participants, total for US participants. Subjective social status is rated on a scale of 1 (low) to 10 (high). Inhibitory control and working memory measures only available in a sub-sample of UK participants. Stroop interference is calculated as the difference between the median response times (milliseconds) of incongruent trials and congruent trials for correct trials only in the Stroop task (a larger interference score is indicative of poorer inhibition). Stroop proportion correct is proportion of trials answered without error. Two error maximum length is the last digit-span a participant got correct before making two consecutive errors in the backwards digit span test. Maximum length is the largest number of digits a participant recalled correctly during all trial in the backwards digit span test.

### Primary analyses

#### SEP predictors of food choice motives (UK sample)

Adjusting for other demographic factors and BMI, lower subjective social status and education level were independently associated with lower health motivation, but household income was not (*p* = 0.052). Results were consistent when the composite measure of education level was used. Results were the same when the multi-item food choice measure was used, with the exception that income became a significant predictor of health motives (*p* = 0.034). In the linear regression model examining weight control motives, lower household income and subjective social status (but not education level) were independently associated with lower weight control motivation. Results were robust across sensitivity analyses. See Table 2 for results in full.

**Table 2:** Linear regression examining demographic and SEP predictors of food choice motives in UK and US samples.

	UK sample (*N* = 4123)				US sample (*N* = 1897)			
	Motives: health R^2^ = 0.08		Motives: weight R^2^ = 0.04		Motives: health R^2^ = 0.05		Motives: weight R^2^ = 0.03	
	B (SE)	*p*	B (SE)	*p*	B (SE)	*p*	B (SE)	*p*
Gender	−0.16 (0.03)	<0.001*	−0.24 (0.03)	<0.001*	−0.14 (0.05)	0.003*	−0.14 (0.05)	0.002*
Ethnicity	0.11 (0.06)	0.051	0.20 (0.06)	0.721	0.06 (0.06)	0.296	0.08 (0.06)	0.181
Age	0.007 (0.001)	<0.001*	−0.01 (0.001)	0.984	0.01 (0.001)	<0.001*	0.002 (0.001)	0.182
BMI	−0.02 (0.003)	<0.001*	0.02 (0.003)	<0.001*	−0.01 (0.003)	<0.001*	0.01 (0.003)	<0.001*
Income	0.02 (0.01)	0.052	0.03 (0.01)	0.03*	−0.01 (0.02)	0.654	0.02 (0.02)	0.411
SSS	0.09 (0.01)	<0.001*	0.07 (0.01)	<0.001*	0.06 (0.01)	<0.001*	0.05 (0.02)	<0.001*
Education	0.22 (0.03)	<0.001*	0.03 (0.03)	0.314	0.17 (0.05)	0.001*	0.11 (0.07)	0.032

Gender reference category is females. Ethnicity reference category is white. Education reference category is lower education. Income ranges from 1–5, lowest to highest quartiles. Motives health and weight reference category is not rating as important. SSS is subjective social status.

*Indicates statistically significant (*p* < 0.05 for primary analyses using UK sample and <0.01 for secondary analyses using US sample).

#### Food choice motives predictors of BMI (UK sample)

Adjusting for demographic variables, being less motivated by health and more motivated by weight control were predictive of higher BMI. See Table 3. Results remained the same in all sensitivity analyses.

**Table 3:** Linear regression examining demographic, SEP and food choice motives predictors of BMI in UK and US samples.

	UK sample (*N* = 4123) R^2^ = 0.09		US sample (*N* = 1889) R^2^ = 0.05	
	B (SE)	*p*	B (SE)	*p*
Gender	−0.53 (0.18)	0.003*	−0.69 (0.34)	0.040
Ethnicity	−0.93 (0.32)	0.004*	−0.63 (0.44)	0.159
Age	0.08 (0.007)	<0.001*	0.02 (0.01)	0.030
Income	0.01 (0.07)	0.882	−0.40 (0.14)	0.004*
SSS	−0.49 (0.06)	<0.001*	−0.29 (0.11)	0.007*
Education level	−0.03 (0.18)	0.876	0.67 (0.37)	0.072
Motives: health	−1.11 (0.10)	<0.001*	−1.37 (0.20)	<0.001*
Motives: weight	1.04 (0.10)	<0.001*	1.35 (0.20)	<0.001*

*G*ender reference category is females. Ethnicity reference category is white. Education reference category is lower education. Income ranges from 1 to 5, lowest to highest quartiles. Motives health and weight reference category is not rating as important. SSS is subjective social status.

*Indicates statistically significant (*p* < 0.05 for primary analyses using UK sample and <0.01 for secondary analyses using US sample).

#### Indirect effects analyses (UK sample)

**Fig. 1:**
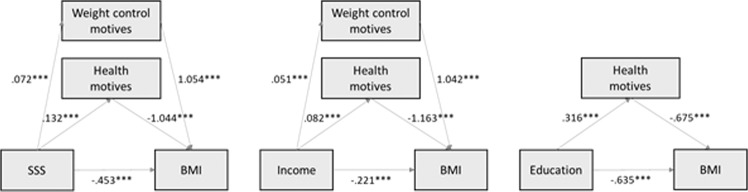
Indirect effect analyses for UK sample. Mediation models between individual measures of SEP and BMI, values are regression coefficients, ***p < 0.001, SSS subjective social status, BMI body mass index.

For the model examining subjective social status and BMI we included both health and weight control motives as parallel mediators (as subjective social status was independently associated with both). We found a negative indirect effect of health motives (−0.138, 95%CI [−0.172; −0.105], explaining 21% of the SEP-BMI association) and a positive indirect effect of weight control motives (0.076, 95%CI [0.052; 0.103], explaining 11% of the SEP-BMI association). We adopted the same approach for household income as income tended to be associated with both food choice motives across the majority of primary and sensitivity analyses. We found a negative indirect effect of health motives (−0.096, 95%CI [−0.127; −0.068], 28% of SEP-BMI association) and a positive indirect effect of weight control motives (0.054, 95%CI [0.030; 0.079], 16% of association). For the model examining education level (categorical) and BMI we included only health motives (single item measure) as education level was not independently associated with weight control motives either in primary or sensitivity analyses. We found a negative indirect effect of health motives (−0.213, 95%CI [−0.290; −0.147], explaining 25% of SEP-BMI association). Figure 1 displays unstandardised regression coefficients for the three mediation models. Results were consistent in all sensitivity analyses.

### Secondary analyses

#### Executive function measures (UK sample)

In the UK sub-sample with measures of executive function (*N* = 3256), poorer inhibitory control (Stroop interference) and working memory (two error maximum length) tended to be weakly associated with higher BMI and lower SEP (rs ranging from 0.001 to 0.095) in unadjusted analyses. In linear regression models, no SEP variables predicted executive function, and no measures of executive function predicted BMI. No executive function measures significantly interacting with food choice motives measures to explain variation in BMI. See online supplementary materials for executive function analyses.

#### Relations between SEP, food choice motives and BMI (US sample)

The US sample (*N* = 1898) was broadly similar to the UK sample in terms of demographic profile, but had a higher proportion of participants with a university degree level of education and above (65% vs. 47%). See Table 1. In unadjusted analyses, results were consistent with the UK sample, whereby there were statistically significant but small positive associations (rs ranging from 0.07 to 0.15) between each measure of SEP and each measure of food choice motives, as well as small negative associations between measures of SEP and BMI (rs ranging from −0.08 to −0.10). See online supplementary materials for results in full. As in the UK sample, higher BMI was associated with lower health motivation (*r* = −0.09) and higher weight control motivation (*r* = 0.08). Similar to the UK sample, in linear regression analyses, lower education level and subjective social status (but not household income) were associated with lower health motives and results remained the same in sensitivity analyses. As in the UK sample, lower subjective social status was significantly associated with lower weight control motives. Household income was not and this pattern of results remain the same across sensitivity analyses. Similar to the UK sample, lower education level was not significantly associated with weight control motives in the main analysis, although in sensitivity analyses in which the multi-item food choice measure was used, this association became significant (*p* = 0.006). See Table 2 for results in full. Similar to the UK sample both lower health motives and higher weight control motives predicted higher BMI when controlling for measures of SEP and demographics (Table 3).

#### Indirect effects analyses (US sample)

We examined whether the association between both education level (composite measure) and subjective social status with BMI were mediated by health motives and weight control motives (single item measures) as both tended to be associated with education level and subjective social status across analyses. We found that both health motives (−0.197, 99%CI [−0.305; −0.105], 24% of association) and weight control motives (0.115, 99%CI [0.035; 0.213], 14% of association) mediated the association between education level and BMI, negatively and positively respectively. We also found that both health motives (−0.103, 99%CI [−0.166; −0.051], 18% of association) and weight control motives (0.085, 99%CI [0.038; 0.140], 15% of association) mediated the association between subjective social status and BMI, negatively and positively respectively. Results were consistent in sensitivity analyses.

### Additional unplanned analyses

#### Analyses limited to multi-item measures of food choice motives

In further sensitivity analyses limited to studies in which only the multi-item health and weight control concern scales were collected results were the same as in the primary analyses. See online supplementary material.

#### Exploratory analyses examining other food choice motives

In studies 1, 2 and 5 participants completed other 1-item measures of food choice motives concerning the motivating influence of mood, convenience, sensory, natural, price, familiarity, environmental, animal welfare and fair trade considerations on food choice. We conducted exploratory analyses to examine if any of the SEP-BMI associations were also mediated by any of these additional motives and found limited evidence. However, we found some evidence that the relationship between lower SEP and higher BMI was mediated by lower SEP participants being less motivated by how natural foods are when making food choices. See online supplementary materials for results in full.

## Discussion

Consistent with previous research in other countries [6–8], across samples of both UK and US adults in unadjusted analyses we found that lower SEP was associated with participants having a higher BMI and reporting being less motivated by health and weight control when choosing foods. However, a novel finding of the present study was the convincing and consistent statistical evidence that cross-sectional associations between lower SEP and higher BMI were in part explained by SEP differences in food choice motives. In particular, among UK adults lower health motives among lower SEP participants explained between 21% and 28% of the association. Similarly, among US adults lower health motives explained between 18% and 24% of this association.

Being more motivated by weight control when making dietary choices were associated with higher BMI. After accounting for health-based motives, weight control motives also mediated some of the SEP and BMI relationship, whereby higher SEP was associated with greater weight control motives and in turn higher BMI. However, this pattern of results was not consistent across all SEP indicators and variance explained tended to be smaller than for health motives (11–16%). Nonetheless, these findings highlight the importance of distinguishing between health and weight control motives when understanding the relationships between food choice motives and BMI. We assume that the positive association between weight control motives and BMI is likely to reflect a greater desire to lose or manage weight among individuals with overweight and obesity and the direction of this relationship may be reversed if examined prospectively. However unsuccessful weight control efforts could contribute to increased weight gain [36–38], so it will be important to understand the potential causal role that any SEP differences in weight control motives has on SEP-BMI associations.

It will now be important to understand SEP differences in health-based food choice motives. For example, lack of financial resources may result in healthiness being deprioritised, as food expenditure has been shown to in part explain SES differences in healthiness of food purchases [23, 39]. Education level and subjective social status were independently associated with health-based food choice motives, which suggests that there may be distinct pathways relating to education (e.g. lack of nutrition literacy) and perceived social standing (e.g. higher psychological distress) that explain the link between lower SEP to lower health motives [40, 41].

We found no convincing evidence that either inhibitory control or working memory explained the cross-sectional association between any indicator of SEP and higher BMI or that relations between food choice motives and BMI were moderated by executive function. These findings may indicate that relations between SEP, executive function and BMI may be better explained by executive function having a causal effect on adult SEP and/or higher BMI having a causal effect on executive function [42], as opposed to SEP patterning of executive function explained SEP-BMI associations. However, we measured only two indices of executive function and it may be the case that other measures (e.g. cognitive flexibility) in part explain links between SEP and BMI [43].

Limitations include reliance on self-reported measures that can be prone to bias. In particular, BMI was based on self-reported weight and height data. Primary analyses also relied on single item measures of food choice motives and this is a limitation. However, results remained the same when limited to a sub-set of studies in which multi-item measures were available. Findings are cross-sectional and therefore we cannot rule out reverse causality, e.g. we presume lower SEP increases risk of higher BMI, but the reverse may also be true [44]. For primary analyses only data on health and weight control food choice motives were available. In exploratory analyses of a limited sub-set of studies we examined other types of food choice motives and found some evidence that being less motivated by how natural foods are may in part explain why lower SEP is associated with higher BMI. However, this was the only significant finding from a number of exploratory models, limited in sample size, and motives were measured with single items. Therefore, caution should be taken when interpreting these analyses. Previous research has shown that participants with low levels of education and income place greater importance on price and familiarity of food than higher educated samples [9] and both importance of price and familiarity explained SES-differences in healthy diet adoption in a UK study [11]. Similarly, it would be informative to examine relative ranking of food choice motives (i.e. the extent to which individuals prioritise health over price) in future research, as in the present studied we relied on absolute ratings of health and weight control food choice motives. The sample was predominantly white and future work would benefit from recruiting more ethnically diverse samples to examine generalisability of findings [45, 46].

## Supplementary information

Online supplementary material

## Data Availability

The study dataset and registered protocol is available on the Open Science Framework repository at https://osf.io/tjgcy/.
