# Supplementary material for: The role of health-based food choice motives in explaining the relationship between lower socioeconomic position and higher BMI in UK and US adults
Source: Int J Obes (Lond). 2022 Jul 21;46(10):1818–24. doi: 10.1038/s41366-022-01190-4 (PMC7613617; doi:10.1038/s41366-022-01190-4)
Supplement: Supplementary file 1 — Online supplementary material [file 41366_2022_1190_MOESM1_ESM.docx]

Online Supplementary Materials for ‘The relationship between lower socioeconomic position and higher BMI is explained by the social patterning of health-based food choice motives in UK and US adults’ by Robinson et al.

*Stroop task information*

Participants saw names of colours presented in varying colours and were asked to indicate the colour of the word by key press as fast as they could whilst trying to restrict errors. The task included congruent trials where the word and the colour it was presented in were the same (e.g. the word ‘blue’ presented in blue text), incongruent trials where colour word and the colour it was presented in were not the same (e.g. the word ‘blue’ presented in red text), and control trials with coloured rectangles in a mixed design. The task included four colours (red, green, blue, black), three colour-stimuli congruency conditions (congruent, incongruent and control), and 7 repetitions for a total of 84 trials (28 congruent, incongruent and control trials). We calculated the median reaction times (RTs) for correct responses in incongruent and congruent trials.

*Backward digit task information*

The task required participants to repeat series of digits (presented visually on screen) of increasing length in reversed order, via key presses. The task was adaptive to performance. If participants made a correct response the subsequent trial became more difficult (addition of a digit), if the participants made an incorrect response the subsequent became easier (removal of a digit). The first trial was a sequence of two digits and the task consisted of 14 trials.

*Supplementary Table 1. Individual study sample sizes*

| Study | Country | Setting | n |
| --- | --- | --- | --- |
| 1 | UK | Fast-food | 868 |
| 2 | UK | Fast-food | 875 |
| 3 | UK | Supermarket | 899 |
| 4 | UK | Portion selection | 1667 |
| 5 | US | Restaurant | 1001 |
| 6 | US | Restaurant | 1090 |

*Supplementary Table 2. Planned data exclusions^[[1]](#footnote-1)^*

- BMI < 18.5 or > = 70 participants
- Weight < 30 or > = 250 participants
- Height < 145 or > = 300 participants
- Equivalised income (UK) > £300,000 or Gross income (US) > $650,000 = 6 participants

| **Study** | **n before exclusion** | **n after exclusion** | **n excluded** |
| --- | --- | --- | --- |
| 1 | 868 | 822 | 46 (5.3%) |
| 2 | 875 | 833 | 42 (4.8%) |
| 3 | 899 | 876 | 23 (2.6%) |
| 4 | 1667 | 1601 | 66 (4.0%) |
| 5 | 1001 | 885 | 116 (11.6%) |
| 6 | 1090 | 1025 | 65 (6.0%) |

*Unplanned data exclusions*

After excluding the above participants (pre-registered), we also identified n=14 participants that did not specify their gender and excluded these participants, resulting in a total sample size of N = 6028 (5.8% of data excluded in total).

*Food choice motives by demographic group*

We originally planned to report metrics for similarity derived from effect sizes observed (e.g. degree of similarity vs. difference between people of lower vs. higher SEP on food choice motives); Cohen’s U3 and probability of superiority; Cohen’s U3 is the expected % of participants with higher SEP expected to be above the average (mean) food choice motives score of participants with lower SEP; Probability of difference is the likelihood that a randomly selected participant with higher SEP have a higher score on food choice motives if compared to a randomly selected participant of lower SEP. However, we now instead report percentages participants from different demographics endorsing each food choice motive as on reflection this is a more direct way of presenting this data (as opposed to making inferences from group means). See supplementary tables 3 and 4.

*Supplementary Table 3. Proportion of participants rating health and weight as ‘important’ when making food choices from 4-item food choice motives questionnaire*

|  | *UK (N = 2475)* | | *US (N = 1898)* | |
| --- | --- | --- | --- | --- |
|  | *Health important* | *Weight important* | *Health important* | *Weight important* |
| *Lower education* | 920 (70%) | 576 (44%) | 224 (64%) | 159 (46%) |
| *Higher education* | 958 (83%) | 530 (46%) | 506 (76%) | 344 (52%) |
|  |  |  |  |  |
| *Lowest income quintile* | 336 (68%) | 209 (42%) | 138 (72%) | 95 (50%) |
| *2^nd^ income quintile* | 355 (71%) | 201 (40%) | 141 (68%) | 94 (45%) |
| *3^rd^ income quintile* | 366 (75%) | 210 (43%) | 149 (70%) | 100 (47%) |
| *4^th^ income quintile* | 407 (82%) | 236 (48%) | 150 (74%) | 107 (53%) |
| *Highest income quintile* | 410 (83%) | 248 (50%) | 152 (76%) | 107 (54%) |
|  |  |  |  |  |
| *Normal weight BMI (18.5-24.9)* | 842 (80%) | 405 (38%) | 285 (74%) | 167 (44%) |
| *Overweight BMI (25-29.9)* | 643 (77%) | 421 (50%) | 237 (75%) | 176 (56%) |
| *Obesity BMI (≥30)* | 393 (67%) | 280 (48%) | 208 (66%) | 160 (51%) |
|  |  |  |  |  |
| *Male* | 895 (74%) | 498 (41%) | 304 (71%) | 203 (471%) |
| *Female* | 983 (78%) | 608 (48%) | 426 (73%) | 300 (52%) |
|  |  |  |  |  |
| *White* | 1708 (75%) | 1004 (44%) | 618 (72%) | 421 (49%) |
| *Not white* | 170 (82%) | 102 (49%) | 112 (73%) | 82 (53%) |

*Lower education denotes below degree/college level. Higher education denotes degree/college level and above*

*Health and weight importance when making food choices scored on a 4-point scale:*

*1 = not at all important, 2 = a little important, 3 = moderately important, 4 = very important*

*‘Important’ = response options 3 and 4*

*Supplementary Table 4. Proportion of participants rating health and weight as important when making food choices from 7-item food choice motives questionnaire*

|  | *UK (N = 1655)* | | *US (N =885)* | |
| --- | --- | --- | --- | --- |
|  | *Health important* | *Weight important* | *Health important* | *Weight important* |
| *Lower education* | 179 (20%) | 131 (15%) | 81 (26%) | 37 (12%) |
| *Higher education* | 253 (33%) | 137 (18%) | 196 (34%) | 94 (16%) |
|  |  |  |  |  |
| *Lowest income quintile* | 82 (26%) | 50 (16%) | 45 (26%) | 24 (14%) |
| *2^nd^ income quintile* | 76 (23%) | 52 (16%) | 56 (32%) | 19 (11%) |
| *3^rd^ income quintile* | 71 (22%) | 50 (16%) | 43 (24%) | 24 (14%) |
| *4^th^ income quintile* | 93 (27%) | 51 (15%) | 64 (36%) | 33 (19%) |
| *Highest income quintile* | 109 (33%) | 64 (19%) | 69 (38%) | 31 (17%) |
|  |  |  |  |  |
| *Normal weight BMI (18.5-24.9)* | 223 (31%) | 112 (16%) | 124 (36%) | 39 (11%) |
| *Overweight BMI (25-29.9)* | 126 (24%) | 87 (16%) | 89 (35%) | 48 (19%) |
| *Obesity BMI (≥30)* | 83 (20%) | 69 (17%) | 64 (23%) | 44 (16%) |
|  |  |  |  |  |
| *Male* | 191 (23%) | 108 (13%) | 126 (30%) | 59 (14%) |
| *Female* | 241 (29%) | 160 (19%) | 151 (33%) | 72 (16%) |
|  |  |  |  |  |
| *White* | 391 (26%) | 248 (16%) | 211 (31%) | 104 (51%) |
| *Not white* | 41 (30%) | 20 (15%) | 666 (33%) | 27 (15%) |

*Lower education denotes below degree/college level. Higher education denotes degree/college level and above*

*Health and weight importance when making food choices scored on a 7-point scale:*

*1: not at all important; 2: not important; 3: not very important; 4: neutral; 5: slightly important; 6: important; 7: very important*

*Important’ = response options 6 and 7*

*Supplementary Table 5. Zero-order associations between measures of SEP, food choice motives and BMI in UK participants (N=4130)*

|  | *Food choice motives health* | *Food choice motives weight* | *Education level (composite)* | *Subjective social status* | *Household income* | *BMI* |  | *Education level low*  *(n=2206)* | *Education level high (n=1924)* | *Low vs. high education* |
| --- | --- | --- | --- | --- | --- | --- | --- | --- | --- | --- |
| *Food choice motives health* | - | .452  p < .001 | .177  p < .001 | .212  p < .001 | .117 p < .001 | -.124  p < .001 |  | M= -0.14 SD= 1.01 | M= -0.17 SD= 0.95 | d = 0.31  p < .001 |
| *Food choice motives weight* | - | - | .040  p = .04 | .117  p < .001 | .073  p < .001 | .084  p < .001 |  | M= -0.02, SD= 0.99 | M= 0.07 SD= 0.99 | d = 0.09  p = .004 |
| *Education level (composite)* | - | - | - | .293  p < .001 | .252  p < .001 | -.107  p < .001 |  | - | - | - |
| *Subjective social status* | - | - | - | - | .398  p < .001 | -.141  p < .001 |  | - | - | - |
| *Household income* | - | - | - | - | - | -.064  p < .001 |  | - | - | - |
| *BMI* | - | - | - | - | - | - |  | M=27.49, SD = 5.94 | M=26.64, SD=5.70 | d = .15  p < .001 |

*Food choices motives measures are z-scored single item only measure. All associations statistically significant (p < .05)*

*Supplementary Table 6. Zero-order associations between measures of SEP, food choice motives and BMI in US participants (N=1898)*

|  | *Food choice motives health* | *Food choice motives weight* | *Education level (composite)* | *Subjective social status* | *Household income* | *BMI* |  | *Education level low*  *(n=660)* | *Education level high (n=1238)* | *Low vs. high education* |
| --- | --- | --- | --- | --- | --- | --- | --- | --- | --- | --- |
| *Food choice motives health* | - | .526  p < .001 | .151  p < .001 | .150  p < .001 | .074  p < .001 | -.089  p < .005 |  | M= -0.17 SD= 1.04 | M= 0.09 SD= 0.95 | d = 0.26  p < .001 |
| *Food choice motives weight* | - | - | .086  p < .001 | .115  p < .001 | .072  p = .002 | .081  p < .001 |  | M= -0.11 SD= 1.01 | M= 0.07 SD= 0.99 | d = 0.18  p = .001 |
| *Education level (composite)* | - | - | - | .370  p < .001 | .341  p < .001 | -.076  p = .001 |  | - | - | - |
| *Subjective social status* | - | - | - | - | .494  p < .001 | -.097  p < .001 |  | - | - | - |
| *Household income* | - | - | - | - | - | -.100  p < .001 |  | - | - | - |
| *BMI* | - | - | - | - | - | - |  | M=28.53, SD=7.19 | M=28.56, SD=7.50 | d = .004  p = .926 |

*Food choices motives measures are z-scored single item only measure. All associations significant (p <.05) with the exception of low vs. high*

*education BMI difference*

*Supplementary Table 7. Zero-order associations between measures of executive function, SEP and BMI in UK participants (N=3256)*

|  | *BMI* | *Education level (z-scored )* | *Income (1-5 quintiles)* | *Subjective social status (1-10)* |
| --- | --- | --- | --- | --- |
| *Stroop*  *interference* | *r = .065 (p < .001)** | *r = -.032 (p = .064)* | *r < .001 (p = .99)* | *r < .001 (p = .989)* |
| *Stroop proportion correct* | *r = -.030 (p = .087)* | *r = .044 (p = .013)* | *r = .033 (p = .058)* | *r =.001 (p = .975)* |
| *Working memory two error max* | *r = -.057 (p = .001)** | *r = .095 (p < .001)** | *r = .045 (p = .010)* | *r = .053 (p = .002)** |
| *Working memory maximum length* | *r = -.046 (p = .009)** | *r =.097 (p < .001)** | *r = .054 (p = .002)** | *r = .046 (p = .009)** |

**indicates statistically significant (alpha value < .01)*

*Supplementary Table 8. Linear regression examining demographic and SEP predictors of executive function measures*

|  | *Inhibitory control*  *Stroop interference* | | *Working memory*  *Two error max length* | |
| --- | --- | --- | --- | --- |
|  | *B (SE)* | *p* | *B (SE)* | *p* |
| *Gender* | *-20.9 (8.2)* | *.010* | *.05 (.06)* | *.484* |
| *Ethnicity* | *49.7 (14.8)* | *.001** | *.14 (.12)* | *.222* |
| *Age* | *3.6 (.34)* | *< .001** | *.003 (.003)* | *.323* |
| *BMI* | *1.4 (0.7)* | *.060* | *-.02 (.006)* | *.008** |
| *Income* | *1.1 (3.2)* | *.730* | *.03 (.03)* | *.216* |
| *SSS* | *-2.1 (2.9)* | *.457* | *.03 (.02)* | *.206* |
| *Education level* | *-5.2 (8.6)* | *.549* | *.16 (.07)* | *.017* |

**indicates statistically significant (alpha value < .01).*

*Gender reference category is female. Ethnicity reference category is white. Education reference category is lower education. Income ranges from 1-5, lowest to highest quartiles. Motives health and weight control reference category is not rating as important. SSS is subjective social status. Results remain same when z-scored measure of education level used.*

*When Stroop proportion correct is used in place of Stroop interference statistical significance of all predictors remains the same. When maximum length error is used in place of two error maximum length statistical significance of predictors remains the same with the exception of age (B=.006, p = .009) and education level (B=.18, p = .004).*

*Supplementary Table 9. Linear regression examining executive function predictors of BMI*

|  | *BMI* | |
| --- | --- | --- |
|  | *B (SE)* | *p* |
| *Model 1* |  |  |
| *Stroop interference* | *.001 (.001)* | *.127* |
| *Two error max length* | *-.134 (.056)* | *.016* |
|  |  |  |
| *Model 2* |  |  |
| *Stroop proportion correct* | *-.026 (.841)* | *.975* |
| *Two error max length* | *-.141 (.061)* | *.021* |
|  |  |  |
| *Model 3* |  |  |
| *Stroop interference* | *.001 (.001)* | *.111* |
| *Maximum length* | *-.129 (.061)* | *.035* |
|  |  |  |
| *Model 4* |  |  |
| *Stroop proportion correct* | *-.026 (.841)* | *.975* |
| *Maximum length* | *-1.41 (.06)* | *.021* |

*All models control for age, gender, ethnicity, income, subjective social status and education level. Alpha value < .01*

Analyses examining whether relationship between food choice motives and BMI is moderated by measures of executive function.

No interaction terms between any measure of food choice motives (weight control or health motives, single item or z-scored measures) or any measure of executive function (Stroop interference, proportion correct, working memory two error maximum length or maximum length) significantly predicted BMI in primary or sensitivity analyses (all ps > .01), indicating no significant evidence that associations between food choice motives and BMI were moderated by measures of executive function.

**Additional Unplanned Exploratory Analyses**

1. **Parallel mediation analyses with multi-item FCQ scales only (health and weight)**
   1. **UK sample – studies 3 and 4 (alpha threshold: 0.05)**

*SEP measure of education categorical (N=2475): results consistent with primary analyses*

| Effect | Mediator | Coefficient | SE | 95%LCL | 95%UCL |
| --- | --- | --- | --- | --- | --- |
| Direct | - | **-0.69** | 0.24 | -1.15 | -0.21 |
| Indirect | Health | **-0.14** | 0.04 | -0.22 | -0.07 |

*SEP measure of income (N=2471): results consistent with primary analyses*

| Effect | Mediator | Coefficient | SE | 95%LCL | 95%UCL |
| --- | --- | --- | --- | --- | --- |
| Direct | - | **-0.293** | 0.082 | -0.456 | -0.131 |
| Indirect | Health | **-0.092** | 0.019 | -0.131 | -0.057 |
|  | Weight | **0.080** | 0.019 | 0.045 | 0.120 |

*SEP measure of SSS (N=2475): results consistent with primary analyses*

| Effect | Mediator | Coefficient | SE | 95%LCL | 95%UCL |
| --- | --- | --- | --- | --- | --- |
| Direct | - | **-0.489** | 0.073 | -0.641 | -0.355 |
| Indirect | Health | **-0.112** | 0.020 | -0.153 | -0.074 |
|  | Weight | **0.106** | 0.019 | 0.070 | 0.146 |

- 1. **US sample – study 6 (alpha threshold: 0.01)**

*SEP measure of education composite (N=1013): results consistent with primary analyses*

| Effect | Mediator | Coefficient | SE | 99%LCL | 99%UCL |
| --- | --- | --- | --- | --- | --- |
| Direct | - | **-0.480** | 0.238 | -1.093 | 0.133 |
| Indirect | Health | **-0.254** | 0.069 | -0.465 | -0.106 |
|  | Weight | **0.164** | 0.056 | 0.041 | 0.334 |

*SEP measure of SSS (N=1013): results consistent with primary analyses*

| Effect | Mediator | Coefficient | SE | 99%LCL | 99%UCL |
| --- | --- | --- | --- | --- | --- |
| Direct | - | **-0.498** | 0.121 | -0.809 | -0.186 |
| Indirect | Health | **-0.127** | 0.036 | -0.239 | -0.049 |
|  | Weight | **0.124** | 0.035 | 0.049 | 0.230 |

1. **Parallel mediation analyses with all additional FCQ dimensions (single item scales) entered simultaneously. Table results show analyses without weight control and health motives included. Asterix indicates if results differ with inclusion^[[2]](#footnote-2)^.**
   1. **UK sample – studies 1 and 2 (alpha threshold: 0.05)**

*SEP measure of education categorical (N=1655): significant negative indirect effect of natural motives, whereby higher SEP was associated with being more motivated by the natural content of food and this predicted lower BMI*

| Effect | Mediator | Coefficient | SE | 95%LCL | 95%UCL |
| --- | --- | --- | --- | --- | --- |
| Direct | - | **-0.6252** | 0.2853 | -1.1849 | -0.0656 |
| Indirect | Mood | 0.0623 | 0.0328 | 0.0064 | 0.1356 |
|  | Convenience | -0.0054 | 0.0148 | -0.0407 | 0.0224 |
|  | Sensory | -0.0105 | 0.0202 | -0.0550 | 0.0276 |
|  | Natural | **-0.2016** | 0.0674 | -0.3448 | -0.0832 |
|  | Price | -0.0349 | 0.0234 | -0.0864 | 0.0034 |
|  | Familiar | -0.0075 | 0.0356 | -0.0790 | 0.0651 |
|  | Environment | -0.0285 | 0.0491 | -0.1326 | 0.0680 |
|  | Animal welfare | 0.0234 | 0.0337 | -0.0360 | 0.0986 |
|  | Fair trade | -0.0569 | 0.0431 | -0.1507 | 0.0177 |

*SEP measure of income (N=1653): significant negative indirect effects of price and natural motives, whereby higher SEP was associated with being more motivated by the natural content of food and less motivated by price, both of which predicted lower BMI*

| Effect | Mediator | Coefficient | SE | 95%LCL | 95%UCL |
| --- | --- | --- | --- | --- | --- |
| Direct | - | -0.0930 | 0.1007 | -0.2906 | 0.1046 |
| Indirect | Mood | -0.0065 | 0.0101 | -0.0287 | 0.0123 |
|  | Convenience | -0.0015 | 0.0047 | -0.0123 | 0.0070 |
|  | Sensory | -0.0041 | 0.0078 | -0.0216 | 0.0099 |
|  | Natural | **-0.0435** | 0.0163 | -0.0786 | -0.0156 |
|  | Price | **-0.0379** | 0.0160 | -0.0725 | -0.0090 |
|  | Familiar | -0.0024 | 0.0080 | -0.0195 | 0.0138 |
|  | Environment | -0.0058 | 0.0106 | -0.0295 | 0.0136 |
|  | Animal welfare | 0.0040 | 0.0066 | -0.0078 | 0.0193 |
|  | Fair trade | -0.0091 | 0.0095 | -0.0329 | 0.0043 |

*SEP measure of SSS (N=1655): significant negative indirect effects of natural motives, whereby higher SEP was associated with being more motivated by the natural content of food and this predicted lower BMI*

| Effect | Mediator | Coefficient | SE | 95%LCL | 95%UCL |
| --- | --- | --- | --- | --- | --- |
| Direct | - | **-0.4147** | 0.0920 | -0.5950 | -0.2343 |
| Indirect | Mood | -0.0081 | 0.0089 | -0.0280 | 0.0078 |
|  | Convenience | -0.0031 | 0.0073 | -0.0194 | 0.0104 |
|  | Sensory | -0.0019 | 0.0071 | -0.0175 | 0.0118 |
|  | Natural | **-0.0610** | 0.0222 | -0.1070 | -0.0203 |
|  | Price | -0.0297* | 0.0161 | -0.0635 | -0.0001 |
|  | Familiar | -0.0013 | 0.0097 | -0.0215 | 0.0175 |
|  | Environment | -0.0079 | 0.0175 | -0.0433 | 0.0265 |
|  | Animal welfare | 0.0046 | 0.0085 | -0.0112 | 0.0235 |
|  | Fair trade | -0.0189 | 0.0143 | -0.0509 | 0.0054 |

*indirect effect of price was non-significant when weight control and health motives were controlled for

- 1. **US sample – study 5 (alpha threshold: 0.01)**

*SEP measure of education categorical (N=885): no significant indirect effects*

| Effect | Mediator | Coefficient | SE | 99%LCL | 99%UCL |
| --- | --- | --- | --- | --- | --- |
| Direct | - | 0.0180 | 0.5425 | -1.3825 | 1.4185 |
| Indirect | Mood | 0.0102 | 0.0281 | -0.0646 | 0.1196 |
|  | Convenience | -0.0190 | 0.0461 | -0.1680 | 0.1206 |
|  | Sensory | -0.0197 | 0.0387 | -0.1485 | 0.0972 |
|  | Natural | -0.0735 | 0.0627 | -0.2996 | 0.0549 |
|  | Price | -0.0014 | 0.0218 | -0.0762 | 0.0787 |
|  | Familiar | -0.0010 | 0.0650 | -0.1836 | 0.1927 |
|  | Environment | 0.0384 | 0.1004 | -0.2360 | 0.3377 |
|  | Animal welfare | 0.0358 | 0.0614 | -0.1186 | 0.2594 |
|  | Fair trade | 0.0213 | 0.0467 | -0.0996 | 0.2088 |

*SEP measure of income (N=885): no significant indirect effects*

| Effect | Mediator | Coefficient | SE | 99%LCL | 99%UCL |
| --- | --- | --- | --- | --- | --- |
| Direct | - | -0.4336 | 0.1869 | -0.9161 | 0.0489 |
| Indirect | Mood | -0.0093 | 0.0150 | -0.0627 | 0.0285 |
|  | Convenience | -0.0308 | 0.0213 | -0.1043 | 0.0102 |
|  | Sensory | 0.0008 | 0.0079 | -0.0237 | 0.0335 |
|  | Natural | -0.0379 | 0.0281 | -0.1283 | 0.0199 |
|  | Price | 0.0097 | 0.0512 | -0.1237 | 0.1486 |
|  | Familiar | 0.0007 | 0.0118 | -0.0353 | 0.0411 |
|  | Environment | 0.0106 | 0.0198 | -0.0405 | 0.0835 |
|  | Animal welfare | 0.0349 | 0.0276 | -0.0162 | 0.1302 |
|  | Fair trade | -0.0027 | 0.0116 | -0.0464 | 0.0319 |

*SEP measure of SSS (N=885): no significant indirect effects*

| Effect | Mediator | Coefficient | SE | 99%LCL | 99%UCL |
| --- | --- | --- | --- | --- | --- |
| Direct | - | -0.1967 | 0.1487 | -0.5807 | 0.1872 |
| Indirect | Mood | -0.0039 | 0.0086 | -0.0358 | 0.0178 |
|  | Convenience | -0.0203 | 0.0165 | -0.0765 | 0.0103 |
|  | Sensory | -0.0002 | 0.0064 | -0.0211 | 0.0242 |
|  | Natural | -0.0408 | 0.0279 | -0.1294 | 0.0225 |
|  | Price | -0.0025 | 0.0324 | -0.0878 | 0.0851 |
|  | Familiar | 0.0000 | 0.0050 | -0.0199 | 0.0168 |
|  | Environment | 0.0089 | 0.0201 | -0.0482 | 0.0729 |
|  | Animal welfare | -0.0074 | 0.0165 | -0.0575 | 0.0409 |
|  | Fair trade | 0.0068 | 0.0137 | -0.0295 | 0.0598 |

1. Participants who failed an attention or did not complete the study were already excluded from original study datasets [↑](#footnote-ref-1)
2. Models including weight control and health motives omitted animal welfare and fair trade motives as mediators (both non-significant across all analyses) due to PROCESS having a maximum number of mediators (10). [↑](#footnote-ref-2)
